# Distinct Virologic Properties of African and Epidemic Zika Virus Strains: The Role of the Envelope Protein in Viral Entry, Immune Activation, and Neuropathogenesis

**DOI:** 10.3390/pathogens14070716

**Published:** 2025-07-19

**Authors:** Ashkan Roozitalab, Chenyu Zhang, Jiantao Zhang, Ge Li, Chengyu Yang, Wangheng Hou, Qiyi Tang, Richard Y. Zhao

**Affiliations:** 1 Department of Pathology, University of Maryland School of Medicine, Baltimore, MD 21201, USA; aroozitalab@som.umaryland.edu (A.R.); chenyu.zhang@som.umaryland.edu (C.Z.);; 2 State Key Laboratory of Molecular Vaccinology and Molecular Diagnostics, National Institute of Diagnostics and Vaccine Development in Infectious Diseases, School of Life Sciences, Xiamen University, Xiamen 361102, Chinahouwangheng@xmu.edu.cn (W.H.); 3 Department of Microbiology, Howard University College of Medicine, Washington, DC 20059, USA; qiyi.tang@howard.edu; 4 Department of Microbiology and Immunology, University of Maryland School of Medicine, Baltimore, MD 21201, USA; 5 Institute of Human Virology, University of Maryland School of Medicine, Baltimore, MD 21201, USA; 6 Institute of Global Health, University of Maryland School of Medicine, Baltimore, MD 21201, USA

**Keywords:** Zika virus (ZIKV), envelope (E) protein, African lineage, Asian lineage, human neural progenitor cell (hNPC)-derived neurosphere, viral entry, cellular immune response, neuropathogenesis, quercetin 3-β-D-glucoside (Q3G), neutralizing antibody

## Abstract

The 2016 Zika virus (ZIKV) epidemic has largely subsided, but a key question remains. How did ZIKV evolve to become a virulent human pathogen compared to the virus of its original discovery? What specific virologic and pathologic changes contributed to increased pathogenicity in humans? Phylogenetic studies have identified two genetically distinct ZIKV, the African and Asian lineages, which differ in their pathogenicity. Previous studies including ours suggest that the envelope (E) protein plays a key role in viral entry, immune activation, and neuropathogenesis. This study aimed to further elucidate virologic and pathogenic differences between these lineages by assessing their ability to bind and replicate in host cells, induce apoptotic cell death, trigger inflammatory responses, and influence human neural progenitor cell (hNPC)-derived neurosphere formation. We compared a historic African ZIKV strain (MR766) with an epidemic Brazilian strain (BR15) and evaluated the effects of the E protein inhibitor quercetin-3-β-O-D-glucoside (Q3G) and an E protein-neutralizing antibody (AbII). Our results revealed distinct virologic properties and that MR766 exhibited stronger inhibition of neurosphere formation due to enhanced viral binding to neuronal SH-SY5Y cells, while BR15 infection triggered a heightened pro-inflammatory cytokine response with reduced viral binding. Chimeric virus studies suggested that the E protein likely influences viral binding, replication efficiency, immune activation, and neuropathogenesis. Notably, Q3G exhibited antiviral activities against both MR766 and BR15, whereas AbII preferentially inhibited MR766. These findings highlight the virological differences between ancestral and epidemic viral strains, as well as the critical role of E protein in viral permissiveness, immune response, and neuropathogenesis, providing insights for developing targeted antiviral strategies.

## 1. Introduction

ZIKV was first isolated in 1947 from a rhesus monkey in Uganda’s Zika Forest [1].

As a result, a ZIKV strain, MR766, was established [1], which has since been widely used in studies of viral pathogenesis and vaccine development [2,3]. Early studies showed that while infected monkeys displayed mild or no symptoms, mice under two weeks old were highly susceptible to infection. In contrast, older mice were largely resistant, likely due to their fully developed blood–brain barrier, which prevented ZIKV from accessing the brain. Subsequent research confirmed that ZIKV is neurotropic with a preference to infect astroglial cells of the central nervous system (CNS), especially embryonic brains [4,5,6]. Although ZIKV is primarily transmitted by *Aedes* mosquitoes to both animals and humans, early ZIKV strains isolated from humans in Africa appeared to cause relatively mild symptoms, even though reactive ZIKV antibodies were present in sera [4,5,7].

Since the initial discovery of ZIKV, it has spread far beyond Africa, with outbreaks documented in Asia and the Americas over the past seventy plus years [2,3,7]. While early infections in Africa and Asia were not associated with severe human disease, continued viral evolution and adaptation led to increased outbreaks and more severe clinical outcomes such as the 2016 ZIKV epidemic. Eventually, ZIKV became a significant human pathogen [8], capable of causing clinical complications such as Guillain–Barré Syndrome (GBS)-like symptoms in adults [9,10] and microcephaly in infants, especially when infection occurs during pregnancy [11,12,13].

Although the 2016 ZIKV epidemic is largely behind us, its impact on global health continues to resonate [8]. A key unresolved question remains: how did ZIKV evolve to become a virulent human pathogen compared to the virus of its original discovery? In other words, what specific virological and pathological changes occurred between the ancestral virus and the pathogenic human-adapted virus that contributed to its increased human pathogenicity? This study aims to address these questions by comparing the virological and pathological differences between the ancestral virus and epidemic strains isolated during the 2016 outbreak in Brazil.

ZIKV is an enveloped, positive-sense, single-stranded RNA (+ssRNA) virus belonging to the genus *Orthoflavivirus* within the *Flaviviridae* family, which includes other medically important arboviruses such as West Nile virus (WNV), Dengue virus (DENV), Japanese encephalitis virus (JEV), and Chikungunya virus (CHIKV) [2]. Like other flaviviruses, ZIKV is transmitted to humans and animals primarily through the bites of infected mosquitoes such as *Aedes aegypti* and *Aedes albopictus*.

ZIKV strains can be broadly classified into two major lineages based on geographic origin and genetic differences [14]. The African lineage includes ancestral strains, while the Asian lineage has been responsible for the major outbreaks in the Pacific and the Americas. Clinically, the Asian lineage is of greater concern due to its association with severe outcomes such as GBS and microcephaly. In contrast, African strains generally cause milder disease in humans. Interestingly, in animal models, African strains tend to be more virulent, often causing more severe disease and higher mortality than their Asian counterparts [15,16].

The ZIKV envelope (E) protein is the major structural protein on the surface of the virion. It plays a central role in viral entry and infection and is considered a key factor in host cell tropism and pathogenesis [3,17,18]. Phylogenetic analyses of E protein sequences between African and Asian ZIKV lineages reveal patterns of divergence that mirror those seen in comparisons of full viral genomes [3,19,20,21]. The E protein exists as a dimer on the surface of the mature virion and is crucial for receptor binding, membrane fusion, and immune recognition. Its ectodomain is composed of three domains—DI, DII, and DIII—along with a stem–transmembrane region [2,22,23]. DI acts as a hinge connecting DII and DIII and allows for structural flexibility. A prominent feature within DI is the glycan loop, which can undergo post-translational N-glycosylation at asparagine residue N154, a modification that may influence viral infectivity [24,25,26]. DII contains a distinctive finger-like structure and a pH-sensitive fusion loop critical for fusion between viral and host membranes during cell entry [27,28]. DIII is important for receptor binding [29,30,31,32]. All these regions could serve as a target for neutralizing antibodies (nAbs) that confer protection against ZIKV infection [33,34]. For instance, the region between the DII and DIII domains is conserved across orthoflaviviruses and plays a crucial role in viral fusion with host membranes; they present an important target for broad-spectrum nAbs against ZIKV infection [3,35].

In this study, we compared the virological and pathological properties of the ancestral African ZIKV strain MR766 with two epidemic Brazilian strains, BeH819015 (BR15) and Paraiba01 (ICD), both of which were isolated during the 2015 Brazil outbreak [11,13,19]. We evaluated their effects on neurosphere-formation-derived human neural progenitor cells (hNPCs) and investigated the role of the E protein in viral entry, host immune response, and neuropathogenesis. Additionally, we examined the effects of a monoclonal neutralizing antibody (AbII) and a natural compound, quercetin-3-β-O-D-glucoside (Q3G), on ZIKV infection and E protein-mediated processes.

## 2. Materials and Methods

### 2.1. Cell Culture and Media

The Human Neural Progenitor ReNcell VM Cell Line is an immortalized cell line (MilliporeSigma, Burlington, MA, USA Cat# SCC008), which was maintained in NSC Maintenance Medium (Millipore, Cat# SCM005), supplemented with 20 ng/mL basic fibroblast growth factor (bFGF, ThermoFisher, Waltham, MA, USA Cat# 13256029) and 20 ng/mL epidermal growth factor (EGF, ThermoFisher Cat# PHG0314). Cell culture vessels for maintaining SCC008 cells were coated with 5 µg/mL Poly-L-ornithine hydrobromide (Sigma, St. Louis, MO, USA P3655) overnight followed by washing with phosphate-buffered saline (PBS) 3 times and coating with 20 µg/mL Laminin (Sigma, L2020) overnight before use. SH-SY5Y (ATCC-CRL-2266) is a human neuroblastoma cell line, which was maintained in Dulbecco’s Modified Eagle’s Medium (DMEM), supplemented with 10% heat-inactivated fetal bovine serum (FBS, Invitrogen, Waltham, MA, USA) and 100 units/mL penicillin plus 100 µg/mL streptomycin.

### 2.2. ZIKV Molecular Clones, Viral Infection, Plaque and Viral Titer Determination

MR766 ZIKV, known as the historical or ancestral strain, the first documented Zika strain, was isolated from a caged rhesus monkey in Uganda’s Zika forest in 1947 [4]. Since this viral strain has undergone extensive passaging over the past seventy plus years in the laboratory, to avoid potential epigenetic modification of the virus, a molecular clone of ZIKV-MR766 was constructed using the infectious subgenomic amplicon (ISA) method, based on the sequence of the ZIKV strain MR766 Uganda 1947-NIID (GenBank accession no. LC002520), as previously described [36]. The BR15 ZIKV strain (BeH819015) was isolated from the blood of a patient in Pará, a northern state of Brazil, in July 2015. This strain was sequenced following a single passage in mosquito C6/36 cells [19,37]. A molecular clone of BR15 was generated based on the BeH819015 sequence (GenBank Accession number: KU365778) using the same ISA strategy.

The two chimeric ZIKV molecular clones, M/B and B/M, were produced as we previously described [38]. The M/B chimeric virus contains the C-prM sequence from the MR766 strain, with the remainder of the viral genome replaced by the corresponding sequence from the BR15 ZIKV molecular clone. In contrast, the B/M chimeric virus has the C-prM sequence from BR15, while the rest of the viral genome is replaced with the corresponding sequence from the MR766 ZIKV molecular clone.

A standard viral plaque assay was used to visualize the plaque size and to quantify the viral titer of MR766 and BR15 with minor modifications [39,40]. Briefly, Vero cells cultured in 48-well plates were infected with tenfold serial dilutions of viral samples for 2 h at 37 °C. Following infection, the cells were overlaid with 0.8% carboxymethylcellulose (CMC) and incubated for 4 days. The cells were then fixed with 3.7% (*w*/*v*) paraformaldehyde in PBS and stained using 0.5% crystal violet dissolved in 20% ethanol. Viral titers were calculated and reported as PFU/mL.

For viral infection assays, cells were seeded into culture plates and incubated overnight at 37 °C with 5% CO_2_ to allow for attachment. The next day, ZIKV was added at a multiplicity of infection (MOI) of 1.0, unless otherwise specified. The cells were incubated with the virus for 2 h at 37 °C, with periodic gentle agitation every 30 min. Following this, the viral inoculum was removed, and the cells were washed twice with sterile PBS. Fresh culture medium was subsequently added, and cells were maintained at 37 °C with 5% CO_2_ for the duration of the experimental timeline. The MOI was calculated by dividing the number of viral particles by the number of cells used for infection. In the experiment assessing the effect of ZIKV infection on neurosphere formation, the MOI was determined based on the number of hNCP cells used to generate the neurospheres.

### 2.3. Adenoviral Construct of MR766 E Protein and Cell Transduction

The adenoviral-E (Adv-E; Adenovirus that carries the gene coding for E protein) construct utilized in this study was custom-designed by ViGene Biosciences (Rockville, MD, USA). The Adv-E construct was propagated in Adeno-X 293 cells (Cat# 632271, Takara, Shiga, Japan), and the viral titer was quantified using the Adeno-X Rapid Titer Kit (Cat# 631028, Takara), which detects the Adenoviral Hexon surface antigen of adenovirus. For Adv transduction, SH-SY5Y cells were seeded at a density of 5000 cells per well in a 96-well plate and incubated overnight at 37 °C with 5% CO_2_ to facilitate attachment. The following day, the cells were transduced with Adv at MOIs of 400, 700, and 1000. Transduced cells were incubated at 37 °C with periodic gentle mixing and assessed at specified time points for further analyses.

### 2.4. Viral Binding Assay

A viral binding assay was performed as we described previously [38] to characterize the attachment of ZIKV to SH-SY5Y cells. Briefly, SH-SY5Y cells were seeded at sub-confluent density. The cell monolayers were washed with cold PBS and incubated at 4 °C for at least 20 min in DMEM containing 2% FBS. Subsequently, the pre-chilled cells were exposed to ZIKV at an MOI of 1 in 1.5 mL of DMEM supplemented with 2% FBS and incubated at 4 °C for 1 h. Following incubation, the viral inoculum was removed, and the cells were washed with DMEM containing 2% FBS to remove any unbound virus. Total cellular RNA was then extracted using TRIzol reagent (Life Technologies, Carlsbad, CA, USA). The RT-qPCR analysis on viral RNA was performed using primers amplifying a conserved region of the ZIKV genome between the NS5 and 3′UTR as described [41]. A housekeeping gene, glyceraldehyde 3-phosphate dehydrogenase (GAPDH), was used as an endogenous control for the measurement of viral bindings.

### 2.5. Measurement of ZIKV Viral Replication

The same real-time RT-PCR method as we described previously to measure ZIKV viral replication over time was used [38]. Briefly, five hundred nanograms of extracted total RNA was used for real-time RT-PCR analysis using the PowerTrack™ SYBR Green Master Mix (Applied Biosystems, Waltham, MA, USA) according to the manufacturer’s instructions. The primer sequences amplify a conserved ZIKV region between the NS5 and 3′UTR. The nucleotide sequences of these primer pairs are ZIKV-F: 5′-AGGATCATAGGTGATGAAGAAAAGT-3′ and ZIKV-R: 5′-CCTGACAACACTAAGATTG-GTGC-3′. Amplification in the QuantStudio™ 3 real-time PCR system involved reverse transcription reaction at 50 °C for 10 min and activation and DNA denaturation at 95 °C for 1 min, followed by 40 amplification cycles of 95 °C for 15 s and 60 °C for 30 s. The mRNA expression (fold-induction) was quantified by calculating the 2^−∆CT^ value, with GAPDH mRNA as an endogenous control.

### 2.6. Measurement of Cell Viability, Cell Death, Apoptosis, and Necrosis

These procedures have been used in our laboratory previously [42,43,44]. Briefly, to measure cell growth, viability, and apoptotic cell death, SH-SY5Y cells were infected by ZIKV or transduced by Adv-E. At the third day post-infection (*p.i.*), cell viability was assessed by MTT assay at the absorbance of 570 nm using a Synergy H1 microplate reader (BioTek Instruments, Winooski, VT, USA), and cell death was quantified by cell counting and trypan blue staining. Cell apoptosis and necrosis were measured using a RealTime-Glo annexin V apoptosis and necrosis assay kit (Promega, Madison, WI, USA) following the manufacturer’s instructions.

All these measurements were conducted in SH-SY5Y cells that were cultured at 5000 cells per well in 100 µL of media in a 96-well plate and incubated overnight at 37 °C with 5% CO_2_ to allow for cell attachment. Three wells containing only the culture medium without cells and viruses were considered as the control. The obtained results were expressed as fold change by normalizing each group’s measured value to the lowest value in the dataset, which was designated as the reference (fold change = 1).

### 2.7. Quantitative RT-PCR Analysis

Total RNA was isolated from SH-SY5Y cells infected with MR766 or BR15 of ZIKV at MOI of 1.0, as well as from uninfected control cells. AT 48 h *p.i.*, total RNA extraction was performed using TRIzol^®^ Reagent (Invitrogen, USA) in accordance with the manufacturer’s protocol. Subsequently, 500 ng of RNA was reverse transcribed into complementary DNA (cDNA) using the High-Capacity cDNA Reverse Transcription Kit (ThermoFisher Scientific, Waltham, MA, USA). The resulting cDNA was utilized as a template for qPCR amplification of cytokine and chemokine genes. A list of primers used in this study to detect various cytokines and chemokines is included in Table S1.

### 2.8. Measurement of hNCP-Derived Neurosphere Formation

The hNCP-derived neurosphere formation assay is a common technique in the study of neurogenesis used to model early neural development [45]. Briefly, hNPCs were seeded into a 60 mm cell culture dish with 1 × 10^6^ cells/dish and cultured overnight. On the second day, a monolayer of cells was infected with ZIKV of indicated MOIs for 2 h, washed with PBS three times, then harvested with Accutase (Sigma, Cat# A6964). Neurospheres were resuspended in a neural differentiation medium mixed with half Advanced DMEM/F12 (ThermoFisher, Cat# 12634010) and half neurobasal-A medium (ThermoFisher, Cat#12349015) supplemented with 1x N2 (ThermoFisher, Cat# 17502048) and 1x B27 supplements (ThermoFisher, Cat#17504044). A total of 5 × 10^5^ cells/well of neurospheres were added onto a 6-well plate and grown under rotation at 90 rpm. Pictures were taken on day 3 or 4 *p.i.* The numbers of neurospheres in each well were counted manually under a microscope, and diameters of neurospheres were measured by BZ-X800 Viewer or ImageJ software 1.54p at day 3 or 4 *p.i.*

### 2.9. Generation of Anti-ZIKV Monoclonal Antibodies Against E Protein

To obtain the anti-ZIKV MAb (AbII), BALB/c mice were immunized with the PRVABC59 (ATCC VR-1843) strain, which was propagated in VERO C1008 cells [Vero 76, clone E6, Vero E6], cells (ATCC CRL-1586) of ZIKV (10^6^ TCID_50_/mouse), emulsified in complete Freund’s adjuvant and subcutaneously boosted twice at 2-week intervals with PRVABC59 in incomplete Freund’s adjuvant. Then, the virus was injected directly into the spleen of the immunized mice. Three days later, splenocytes from the immunized mice were fused with Sp2/0-Ag-14 according to hybridoma technology. Antibodies against PRVABC59 in the hybridoma supernatant were screened by an enzyme-linked immunosorbent assay (ELISA) against the PRVABC59 E antigen. Positive wells were cloned at least twice. Finally, ascitic fluid produced from a single clone of positive cells was purified by protein A chromatography and named AbII.

### 2.10. Molecular Dynamic (MD) Simulations

The immune complex structure used for molecular dynamics simulations was generated using predictions from the AlphaFold3 online platform (https://alphafoldserver.com/, accessed on 1 January 2025) [45]. Each predicted model underwent a 120 nanosecond (ns) MD simulation using the GROMACS 2024.2 software package [46,47] with the CHARMM36 force field [48]. The system was solvated in a cubic box with a minimum distance of 1.0 nm between the protein surface and the box edge, using the TIP3P water model. Sodium (Na^+^) and chloride (Cl^−^) ions were added to neutralize the system and to achieve a final ion concentration of 150 mM. Energy minimization was performed with 1000 steps of the steepest descent until the maximum force on any atom was less than 1000 kJ/mol/nm. After energy minimization, the system was equilibrated in two stages: (1) a 200 picosecond (ps) NVT (constant number of particles, volume, and temperature) simulation at 310 K using the Nose–Hoover thermostat and (2) a 1-nanosecond (ns) NPT (constant number of particles, pressure, and temperature) simulation at 1 bar using the Berendsen barostat. The production of MD simulation was carried out under NPT conditions at 310 K and 1 bar using the Parrinello–Rahman barostat. Long-range electrostatic interactions were calculated using the Particle Mesh Ewald (PME) method with a 1.2 nm cutoff for Coulomb interactions, while van der Waals interactions were truncated at 1.2 nm. The LINCS algorithm was employed to constrain all bond lengths, enabling the use of a 2-femtosecond (fs) time step. Trajectory data were saved every 10 ps for subsequent analysis. The simulation trajectories analysis, including root mean square deviation (RMSD) and interface interaction evaluations, was performed using the Discovery Studio 2024 software package. Visualization and additional trajectory analysis were conducted using molecular dynamics (VMD) [49] and MDAnalysis [50].

### 2.11. Q3G and AbII Treatment

Q3G, also referred to as isoquercitrin, was obtained from Sigma-Aldrich (St. Louis, MO, USA) and prepared as stock solutions in sterile dimethyl sulfoxide (DMSO, Sigma-Aldrich, USA). The monoclonal AbII, generated as described in Section 2.9, was used in all experiments with a final dilution of 1:320 from a stock concentration of 930 µg/mL. This dilution was selected based on our initial dose-dependent curve of the AbII against the PRVABC59 strain. A growth culture medium containing 0.2% DMSO served as the vehicle control.

For viral infection experiments involving AbII or Q3G, these agents were added alongside the virus during the initial 2 h infection period. Following PBS washes, AbII or Q3G were re-added to the culture medium for the duration of the experimental timeline. To measure viral binding in cells treated with Q3G, it was added at the indicated concentration and incubated for 1 h during at 4 °C with ZIKV at MOI of 1.0. For the treatment with AbII, AbII was added at the final 1:320 dilution for 1 h during at 4 °C with ZIKV at an MOI of 1.0.

To evaluate the effect of AbII and Q3G on the survival of Adv-transduced SH-SY5Y cells, cells were transduced by Adv-E derived from MR766 at the indicated MOI and were treated by a range of different concentrations of AbII or Q3G. Cell viability was assessed after 72 h using the MTT assay. The 50% cytotoxic concentration (CC_50_) was determined through nonlinear regression analysis, using a sigmoidal concentration–response curve (variable slope; GraphPad Prism; San Diego, CA, USA).

### 2.12. Statistical Analysis

Unless indicated, a two-tailed and paired Student *t*-test was used for a pair-wise comparison of data, using GraphPad Prism software 10 (GraphPad Software, San Diego, CA, USA). Two-way ANOVA analysis was used to analyze results generated for neurosphere experiments using Prism ver. 10. A difference is considered statistically significant if *p* ≤ 0.05 (*), *p* ≤ 0.01 (**), *p* ≤ 0.001 (***), or *p* ≤ 0.0001 (****) according to conventional definitions.

## 3. Results

### 3.1. Comparison of ZIKV Infection on hNPC-Derived Neurosphere Formation and Host Cellular Immune Response Between the Ancestral MR766 and Epidemic BR15 and ICD Strains

The hNPC-derived neurosphere formation assay is a commonly used technique for modeling early neurogenesis in vitro [45]. Neurospheres are free-floating, three-dimensional cellular aggregates generated from hNPCs and have been widely employed to study neural development [51,52,53]. Given that ZIKV infection is known to disrupt early neural development and contribute to microcephaly [11,12,13] this assay was used as a relevant and quantifiable model to evaluate the impact of ZIKV infection in a heterogeneous neural cell population.

**Figure 1:**
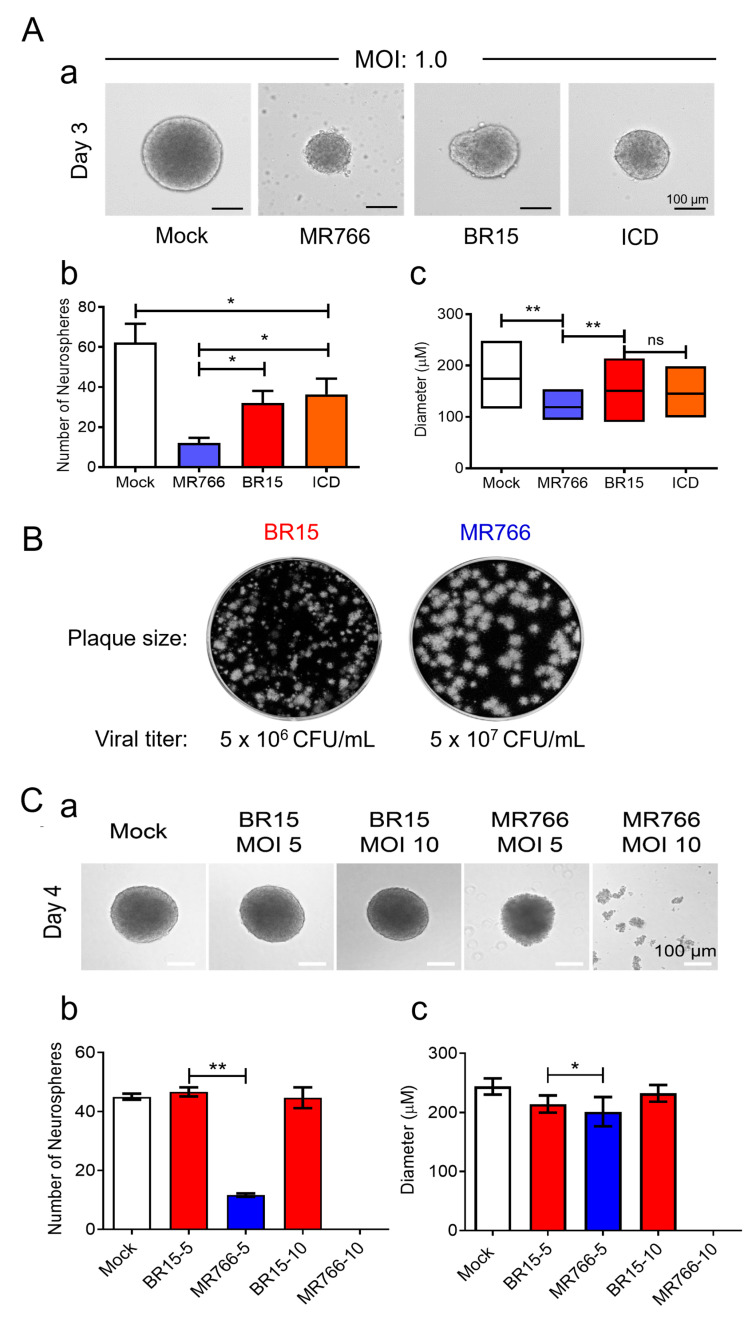

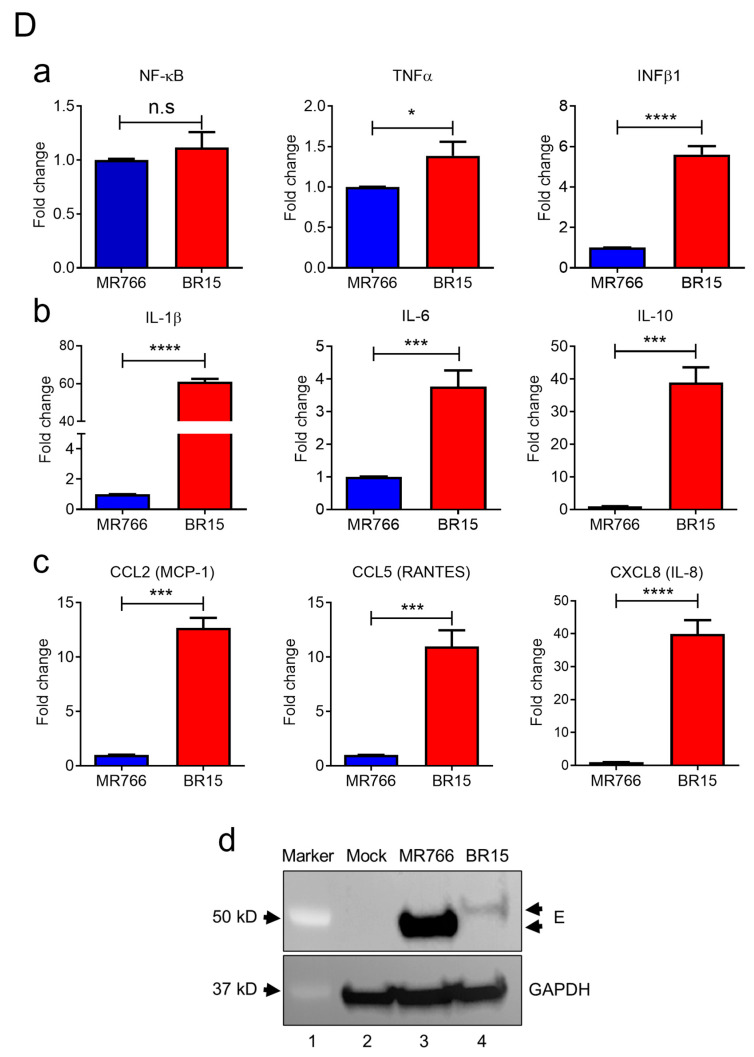
Effect of ancestral MR766 and epidemic BR15 and ICD ZIKV infection on neurosphere formation and cellular immune response. (**A**) Effect of ZIKV infection on NPC-derived neurosphere formation. Representative images of neurospheres (**a**), quantification of neurosphere formation (**b**), and neurosphere diameters (**c**). The NeNcell VM hNPCs were infected with MR766, BR15, and ICD at an MOI of 1.0 along with an uninfected (Mock) control. The neurosphere formation was observed at three days *p.i.* The total numbers of neurospheres in each of the 6-well plates were counted manually under the microscope, and the diameters of neurospheres were measured by ImageJ software. The upper, middle, and lower lines of the box in (**c**) indicate the maximum, mean, and minimum values, respectively. Data are presented as mean ± SD from three independent experiments. (**B**) MR766 displays a larger plaque size and higher viral titers than BR15. Images show an example of different sizes of infectious plaques developed by MR766 and BR15 after infection to Vero76 cells in the plaque-forming assay. Viral titers were determined using the standard plaque-forming assay, as previously described [44], and measured as plaque-forming units per milliliter (PFU/mL). (**C**) Contrasting effect of MR766 and BR15 on the formation of the neurosphere with an increased MOI. Representative images of neurosphere formation are shown at day 4 *p.i.* by MOI 5 and 10 of MR766 and BR15 ZIKV strains (**a**). Additional numbers of neurospheres are shown in Figure S1A. Quantification of neurosphere formation (**b**) and neurosphere diameters (**c**). Note that infection with MR766 at MOI 10 results in complete destruction of neurosphere formation. Scale bar in neurosphere images represents 100 µm. (**D**) Relative cellular immune response to MR766 in comparison to BR15 infection to SH-SY5Y cells. Neuroblastoma SH-SY5Y cells were infected with MR766 or BR15 with an MOI of 1.0. Infected cells were collected at 48 h *p.i.* The levels of NF-kB, TNFα, and INFβ1 (**a**), IL-1 and IL-6 cytokines (**b**), and CXC chemokine (**c**) productions were measured by qRT-PCR, and production of the E proteins of MR766 and BR15 was confirmed by Western blot analysis (**d**). Note that the increased size of BR15 E protein is likely due to N154-glycosylation as indicated by arrows, whereas MR766 is not glycosylated at N154, as reported previously [3,26,56]. To compare the relative abundance of cellular immune responses between MR766 and BR15, the level of immune response to MR766 was set to 1. All experiments were carried out at least two to four times. Statistical significance is indicated as * for *p* < 0.05, ** for *p* < 0.01, *** for *p* < 0.001, and ***** for *p* < 0.0001. n.s., not significant.

The objective of this experiment was to compare the effects of ZIKV infection on neurosphere formation between the ancestral African strain MR766 and the epidemic Brazilian strains BeH819015 (BR15) and Paraiba01 (ICD) [11,13,19]. Neurospheres were generated using the immortalized hNPC cell line SCC008 [45]. Before neurosphere formation, hNPCs were infected with MR766, BR15, or ICD at an MOI of 1.0, alongside a mock control. Neurosphere numbers and diameters were quantified using microscopic imaging on day 3 *p.i.* (Figure 1A).

In the mock control group, typical spherical neurospheres developed in the absence of ZIKV infection. In contrast, all ZIKV-infected cultures showed reduced neurosphere sizes and abnormal morphology. Notably, MR766-infected cultures displayed significant cellular debris, which was largely absent in BR15- or ICD-infected neurospheres (Figure 1A-a). On average, the mock control yielded 62 ± 13 neurospheres with a mean diameter of 174 ± 30 µm (Figure 1A-b,c). MR766-infected cultures exhibited a marked 80% reduction in neurosphere number (13 ± 4) and a 30% decrease in diameter (120 ± 15 µm). BR15 and ICD infections resulted in a more moderate reduction in neurosphere number (42–50%) and diameter (14–16%). These differences between MR766 and the epidemic strains were statistically significant (*p* < 0.01). Since BR15 and ICD showed similar effects, only BR15 was used for subsequent experiments.

To further explore the virological differences between MR766 and BR15, we first examined plaque morphology and viral titers (Figure 1B). MR766 formed larger plaques and yielded viral titers approximately one log higher than BR15. We next assessed the impact of higher viral loads on neurosphere formation by increasing the MOI from 1.0 to 5.0 and 10.0 and extending the observation period to day 4 *p.i.* (Figure 1C). BR15-infected hNPCs exhibited similar neurosphere morphology, size, and number at both MOIs. In contrast, MR766-infected cultures showed a significant reduction in neurosphere number at both MOIs in comparison with the mock control, and complete destruction of neurospheres was observed at MOI 10 by day 4 (Figure 1C). Additional numbers of neurospheres are shown in Figure S1A.

To investigate potential differences in host cellular immune responses triggered by MR766 and BR15, we measured the expression of pro-inflammatory cytokines, chemokines, and NF-κB, a major transcription factor involved in immune regulation [54]. The SH-SY5Y neuroblastoma cell line, which is permissive to ZIKV infection [55], was infected with MR766 or BR15 at MOI 1.0, and RNA was extracted at 48 h *p.i.* for qRT-PCR analysis of seventeen immune markers. No significant differences were observed in NF-κB expression (Figure 1D-a) or in eight additional cytokines and chemokines (Figure S1B). BR15 infection induced a modest but statistically significant increase in TNF-α expression (*p* < 0.05) and a significantly higher expression of IFN-β1 (Figure 1D-a), IL-1β, IL-6, and IL-10 (Figure 1D-b), as well as the chemokines CCL2 (MCP-1), CCL5 (RANTES), and CXCL8 (IL-8) (Figure 1D-c), compared to MR766-infected cells.

In summary, all ZIKV strains impaired neurosphere formation, but MR766 caused the most pronounced reductions in both neurosphere number and size. BR15 and ICD had milder yet comparable effects. Furthermore, BR15 infection elicited a stronger pro-inflammatory cytokine and chemokine response than MR766 in SH-SY5Y neural cells.

### 3.2. Correlation of the ZIKV E Protein with Viral Attachment, Replication, and hNPC-Derived Neurosphere Formation

**Figure 2:**
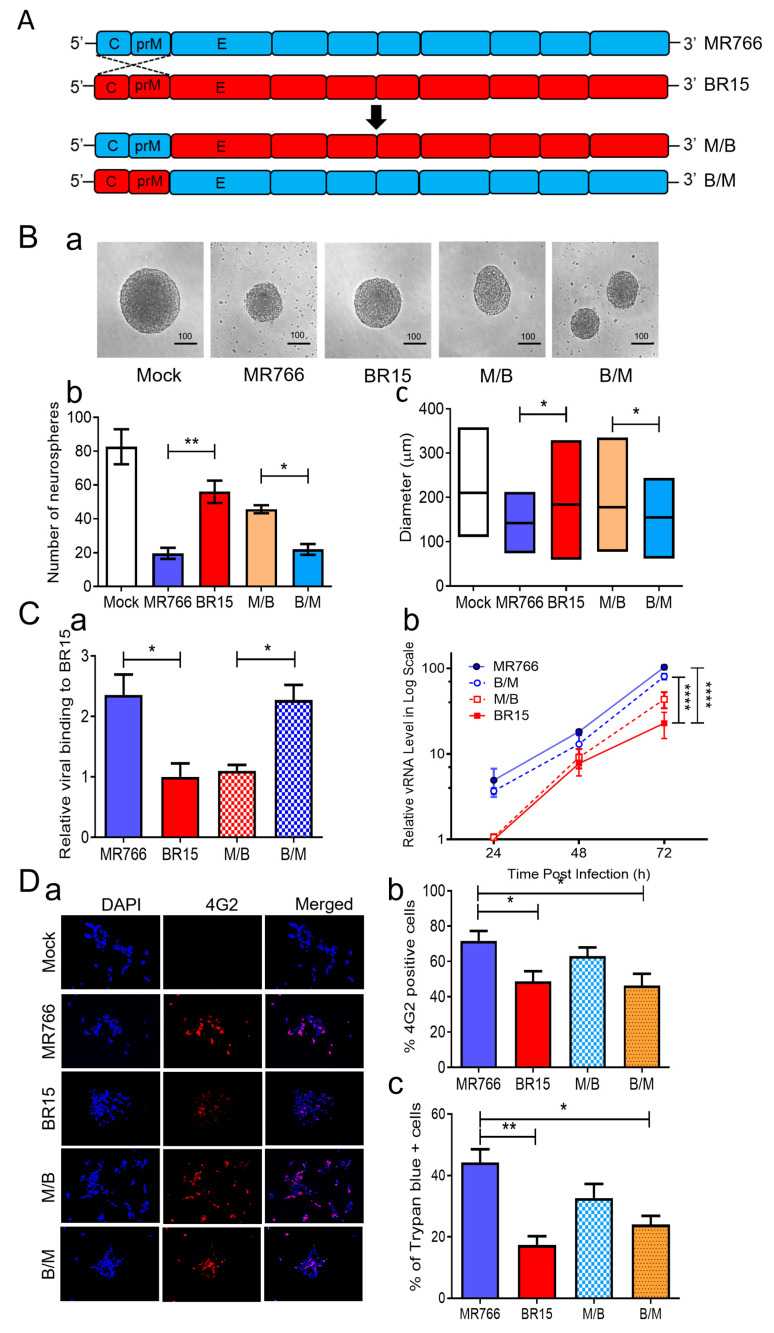

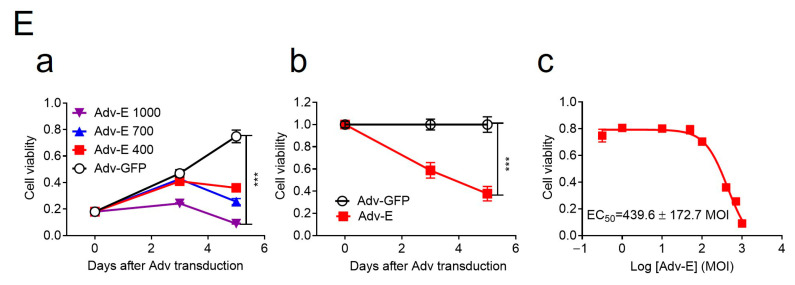
Correlation of the ZIKV E protein with viral attachment and hNPC-derived neurosphere formation. (**A**) Schematic representation of chimeric ZIKV molecular clones and their parental clones. The chimeric viruses were generated by exchanging genomic regions between the MR766 and BR15 ZIKV molecular clones at the prM and E protein junction as previously described [42]. (**B**) Effect of chimeric ZIKV infection on neurosphere formation. Representative images of neurosphere development were taken at three days *p.i.* with ZIKV strains MR766 and BR15 and chimeric strains (M/B and B/M) or uninfected controls (Mock) (**a**). Additional numbers of neurospheres are shown in Figure S2. Quantification of neurosphere number (**b**) and size (**c**) in infected and uninfected groups. Quantitative results are presented as mean ± SD. Scale bar in neurosphere images represents 100 µm. Statistical significance: * *p* < 0.05; ** *p* < 0.01; *** *p* < 0.001; **** *p* < 0.0001. (**C**) Comparison of viral binding (**a**) and replication (**b**) between MR766, BR15, and chimeric viruses. Viral binding was quantified by measuring cell-associated vRNA at one-hour *p.i.*, with GAPDH serving as the endogenous control. Results are presented as mean ± SD from four independent experiments. ZIKV replication was assessed by RT-qPCR over the indicated time points and expressed in log-scale. (**D**) Comparison of viral permissiveness and cell death between MR766, BR15, and chimeric viruses. Viral infection was evaluated using the anti-E mAb 4G2 at 48 h *p.i.* (**a**) with quantification of the fluorescence intensity (**b**) and ZIKV-induced cell death (**c**) as measured by the Trypan blue exclusion assay at 72 h *p.i.* (**E**) Adv-E protein of MR766 induces cell death and reduces cellular growth of human neuroblastoma HS-SY5Y cells. (**a**) Dose-dependent reduction in cell growth and proliferation induced by Adv-E over 5 days, assessed using the MTT assay. SH-SY5Y cells were transduced with Adv-E at MOIs of 400, 700, and 1000, while Adv-GFP was used as the control group. (**b**) Effect of Adv-E (MOI 1000) on cell growth as compared with the Adv-GFP control overtime. (**c**) Determination of the EC_50_ of Adv-E-induced cell death in SH-SY5Y cells. All experiments were carried out at least two to four times.

Given the clear differences in neurosphere formation caused by the ancestral MR766 and epidemic BR15 strains (Figure 1), we next sought to identify virological determinants responsible for these effects. Prior studies have shown that the structural proteins (C-prM-E) of ZIKV are critical for initiating infection and exhibit differential abilities in viral attachment [41]. Our previous analyses of chimeric viruses, generated by swapping the C-prM region between MR766 and BR15, suggested that the E protein correlates with viral attachment and replication, while the C-prM region influences cell permissiveness and ZIKV-induced cytopathic effects in neural and epithelial cell lines [38]. To investigate whether the structural proteins, particularly the E protein, account for the differences in neurosphere formation, we compared the effects of these previously characterized chimeric viruses with their parental MR766 and BR15 strains [38,41]. In the M/B and B/M chimeric viruses, the C-prM region of the structural proteins was swapped between MR766 (blue) and BR15 (red), while each virus retained its own E protein (Figure 2A). This design allowed us to directly assess the functional contribution of the E protein of structural proteins from each strain to neurosphere formation by holding the E protein and the rest of the genome constant while varying the associated C-prM region.

Neurospheres were generated using the same protocol described in Figure 1. hNPCs were infected with MR766, BR15, M/B, or B/M at an MOI of 1.0, and the neurosphere number and diameter were measured on day 3 *p.i.* In the mock group, neurospheres appeared intact, large, and spherical. In contrast, MR766- and B/M-infected cells, both containing the MR766 E protein, formed significantly fewer and smaller neurospheres with greater background cell debris, suggestive of cell death (Figure 2B). BR15 and M/B viruses, both carrying the BR15 E protein, resulted in a relatively preserved neurosphere morphology and number. These data suggest that the E protein primarily accounts for the differential effects on neurosphere formation between MR766 and BR15.

To determine if these viruses behaved similarly as we reported in other human cells [38], we examined their attachment, replication, infection efficiency, and cytopathic effects in SH-SY5Y cells. First, we assessed viral binding by infecting SH-SY5Y cells at MOI 1.0 for 1 h at 4 °C, followed by qRT-PCR quantification of cell-associated viral RNA. MR766 and B/M viruses exhibited significantly higher attachment compared to BR15 and M/B (Figure 2C-a), confirming the role of the MR766 E protein in enhanced viral binding. Next, we evaluated viral replication kinetics. SH-SY5Y cells were infected at MOI 1.0 for 2 h at 37 °C, and intracellular viral RNA was measured over 3 days *p.i.* MR766 and B/M consistently showed higher vRNA levels compared to BR15 and M/B (Figure 2C-b), aligning with our prior findings that the E protein governs viral attachment and replication [38].

To further explore the relationship between viral genotype and infection efficiency, we stained SH-SY5Y cells at 48 h *p.i.* using the flavivirus-specific anti-E monoclonal antibody 4G2 [57]. Quantification of infected cells (Figure 2D) revealed that infection levels correlated more strongly with the C-prM region than the E region. Specifically, MR766-infected cells showed the highest infection rate (71.6 ± 9.4%), similar to M/B (63.0 ± 8.54%), both carrying MR766’s C-prM. Conversely, BR15 and B/M infections resulted in lower infection rates (48.6 ± 7.02% and 46.3 ± 6.67%, respectively), consistent with the presence of BR15’s C-prM. These findings are consistent with our earlier findings supporting the role of C-prM in viral permissiveness [38].

We next assessed cytopathic effects using Trypan blue exclusion. As expected, cell death also correlated with the C-prM region. MR766 and M/B induced higher levels of cell death (44.3 ± 7.37% and 32.7 ± 4.02%, respectively) than BR15 and B/M (17.3 ± 3.03% and 24.0 ± 4%, respectively). However, discrepancies in cytotoxicity between MR766 and M/B or between BR15 and B/M suggest that the E protein may also contribute to cell death [58]. To further test the cytotoxic role of the E protein, we generated an adenoviral construct (Adv-E) expressing the MR766 E protein and transduced SH-SY5Y cells at increasing MOIs. Cell viability, measured by an MTT assay, decreased in a dose-dependent manner (Figure 2E-a), with maximal cell death observed at MOI 1000. Cell counts relative to Adv-GFP controls showed time-dependent reductions in Adv-E-transduced cells (Figure 2E-b), further indicating E protein-induced cytotoxicity. The half-maximal effective concentration (EC_50_) of Adv-E was determined to be 439.6 ± 172.7 (Figure 2E-c).

In summary, comparisons between chimeric and parental ZIKV strains revealed that the E protein plays an important role in neurosphere disruption, primarily through enhanced viral attachment and replication, which in turn contributes to cytotoxicity and impaired neurosphere formation. The C-prM region influences infection efficiency and permissiveness, with both structural components contributing to the overall observed phenotype.

### 3.3. Q3G Suppresses the Impacts of MR766 and BR15 on Neurosphere Formation Through Disruption of E Protein-Mediated Virological and Cellular Immune Effects

**Figure 3:**
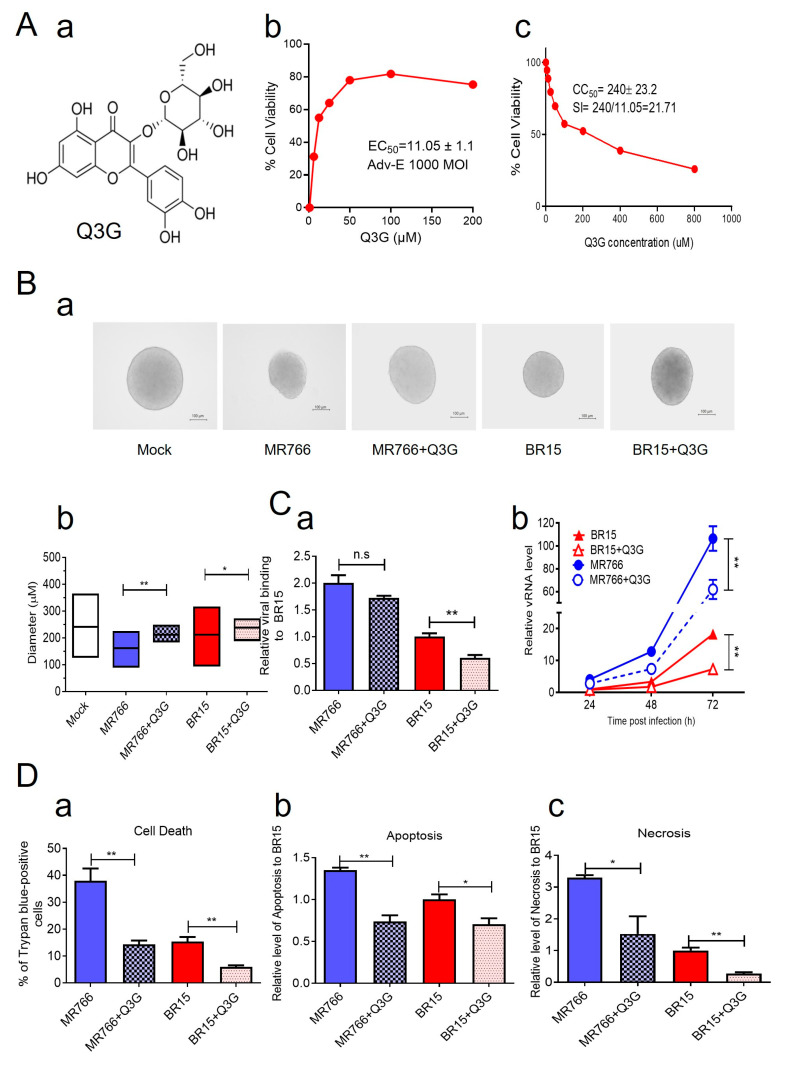

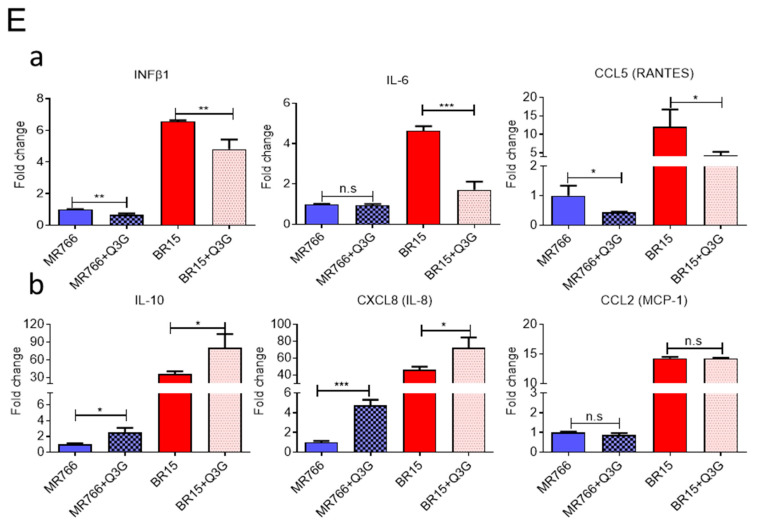
Q3G suppresses both MR766 and BR15 ZIKV-mediated effects including viral attachment, replication, cellular immune response, and neurosphere formation. (**A**) Chemical structure of Q3G (**a**) is a quercetin glycoside where a glucose molecule is attached to the 3-position of the quercetin structure via a β-glycosidic linkage. This attachment forms a mono-glucoside, with the glucose molecule directly connected to the flavonoid core. (**b**) Determination of EC_50_ value of Q3G against Adv-E-induced cell death at an MOI of 1000. (**c**) Determination of the CC_50_ and calculation of SI of Q3G. SH-SY5Y cells were incubated for 72 h with Q3G at indicated concentrations. (**B**) Images of neurospheres in Q3G-treated and untreated groups were taken at day 3 *p.i.* (**a**), and the size of the neurosphere (**b**) was quantified in the same way as described in Figure 1. Scale bar in neurosphere images represents 100 µm. Note that Q3G promotes aggregation of neurospheres. As a result, we were unable to accurately represent the number of neurospheres. (**C**) Q3G reduces viral binding (**a**) and intracellular viral replication (**b**). (**D**) Induction of cell death as measured by Trypan blue (**a**), apoptosis (**b**), and necrosis (**c**) as measured by a RealTime-Glo annexin V apoptosis and necrosis assay kit (Promega). (**E**) Q3G suppresses pro-inflammatory (**a**) but promotes production of anti-inflammatory cytokines and chemokines (**b**). SH-SY5Y cells were infected with BR15 or MR766 ZIKV strains at an MOI of 1.0 in the presence or absence of Q3G (12.5 μM). The same methods and timeframe as described in Figure 1 were used to measure viral binding at one-hour *p.i.*, intracellular viral replication over a time period of 72 h *p.i.*, cellular immune response at 48 h *p.i.*, and measurement of neurosphere formation at 3 days *p.i.* Results shown for intracellular viral replication represent SD from four independent experiments. ZIKV-induced apoptotic cell death was first quantified by the Trypan blue exclusion assay. Cell apoptosis and necrosis were then measured using a RealTime-Glo annexin V apoptosis and necrosis assay kit (Promega). The results are from three independent experiments. Statistical significance is indicated as * for *p* < 0.05, ** for *p* < 0.01, and *** for *p* < 0.001. n.s., not significant.

Q3G, also known as isoquercitrin, is a flavonoid with well-documented antiviral and anti-inflammatory properties against various viruses (Figure 3A-a) [59]. It has also been reported to inhibit ZIKV infection by targeting the early stages of infection and preventing viral internalization during the entry process [60,61]. Based on this information, we chose to evaluate the effects of Q3G and hypothesized that its treatment would reduce viral attachment and replication of both the ancestral MR766 and epidemic BR15 strains, thereby mitigating cytopathic effects and preserving the integrity of hNPC-derived neurospheres.

We first assessed whether Q3G can interfere with E protein activity. SH-SY5Y cells were transduced with Adv-E, an adenoviral construct expressing the MR766-derived E protein. Q3G treatment significantly restored cell viability in a dose-dependent manner, with an EC_50_ of 11.05 ± 1.1 µM (Figure 3A-b). Q3G exhibited low cytotoxicity, with a 50% cytotoxic concentration (CC_50_) of 240 µM, yielding a therapeutic index (TI) of 21.71 (Figure 3A-c), indicating a moderate safety margin for antiviral efficacy.

We next examined whether Q3G could protect neurosphere formation during ZIKV infection. hNPC-derived neurospheres were infected with MR766 or BR15 (MOI 1.0), with or without Q3G. Q3G treatment improved neurosphere integrity (Figure 3B-a) and significantly increased neurosphere size in both the MR766 + Q3G group compared to the MR766 group (211.4 ± 19.7 µm vs. 162 ± 35.7 µm) and the BR15 + Q3G group compared to the BR15 group (211.31 ± 59.9 µm vs. 238.7 ± 19.5 µm), in comparison to their respective untreated controls (Figure 3B-b). Interestingly, Q3G-treated cultures displayed frequent neurosphere fusion, forming aggregates that precluded accurate quantification of neurosphere numbers. Thus, only the diameters of intact neurospheres were measured.

To further assess Q3G’s antiviral effects, we examined its impact on viral attachment and replication in SH-SY5Y cells. Q3G modestly reduced viral attachment of both strains, although the difference in MR766 attachment with or without Q3G was not statistically significant (Figure 3C-a). However, Q3G significantly reduced intracellular viral RNA levels at 24 h *p.i.* in both MR766 and BR15 infections (Figure 3C-b), indicating that Q3G suppresses ZIKV replication after viral entry.

We then assessed whether Q3G could attenuate ZIKV-induced cytotoxicity. Trypan blue staining revealed that Q3G treatment significantly reduced ZIKV-induced cell death, with average decreases of 62.2% and 60.9% in MR766- and BR15-infected cells, respectively (Figure 3D-a). To characterize the mode of cell death, we performed real-time Annexin V apoptosis and necrosis assays. Both MR766 and BR15 induced apoptosis and necrosis in SH-SY5Y cells, and Q3G treatment reduced apoptosis by 45.5% (MR766) and 29% (BR15), and necrosis by 53.7% (MR766) and 70% (BR15), compared to their respective controls (Figure 3D-b,c).

Because BR15 infection triggered stronger innate immune responses than MR766 in SH-SY5Y cells (Figure 1D), we examined whether Q3G modulates these responses. Under the same infection conditions, Q3G treatment significantly reduced production of IFN-β1, IL-6, and CCL5 (RANTES) in both MR766- and BR15-infected cells (Figure 3E-a). In contrast, IL-1β and CCL2 (MCP-1) levels remained unchanged by Q3G, suggesting that these factors may be induced by other viral components other than E protein (Figure 3E-b and Figure S2). Interestingly, Q3G treatment increased anti-inflammatory IL-10 and chemokine CXCL8 (IL-8) levels in both groups (Figure 3E-b), although BR15-infected cells consistently exhibited higher overall cytokine levels than MR766-infected cells, even in the presence of Q3G. Other cytokines or chemokines that did not respond to Q3G are listed in Figure S3.

Collectively, these findings demonstrate that Q3G mitigates the deleterious effects of ZIKV on neurosphere formation, likely through interference with E protein-mediated viral entry and replication, suppression of virus-induced apoptosis and necrosis, and modulation of pro-inflammatory and anti-inflammatory cytokine responses. These data suggest that Q3G holds promise as a therapeutic candidate for preserving neurodevelopmental integrity during ZIKV infection.

### 3.4. AbII Preferentially Neutralizes MR766 Activities over BR15

To identify Abs targeting ZIKV E protein, we screened candidates of mAbs and identified AbII, which was generated against the ZIKV PRVABC59 strain isolated in 2015 from an asymptomatic individual in Puerto Rico [62]. Dose–response testing using the Adv-E expression system in SH-SY5Y cells confirmed that AbII selectively neutralizes MR766 E protein activity, with a half-maximal inhibitory concentration (IC_50_) of 2.95 ± 0.25 µg/mL (Figure S4A).

**Figure 4:**
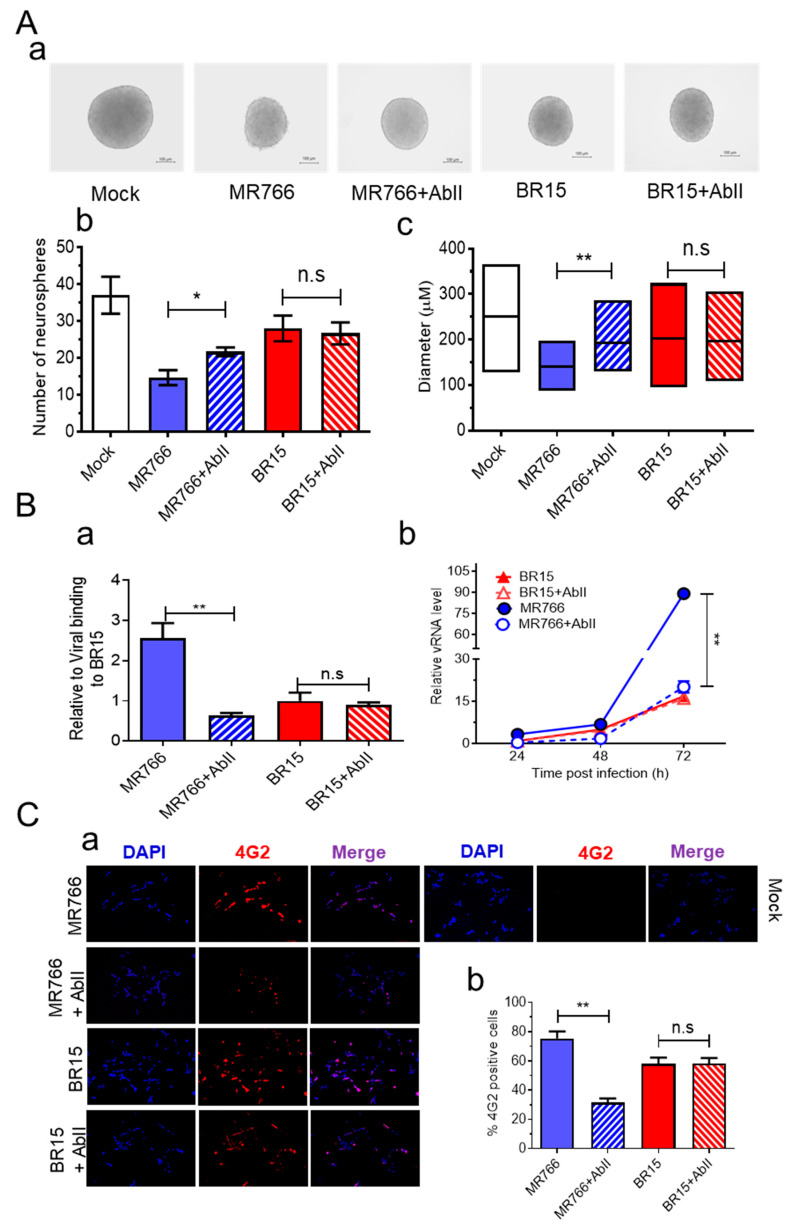

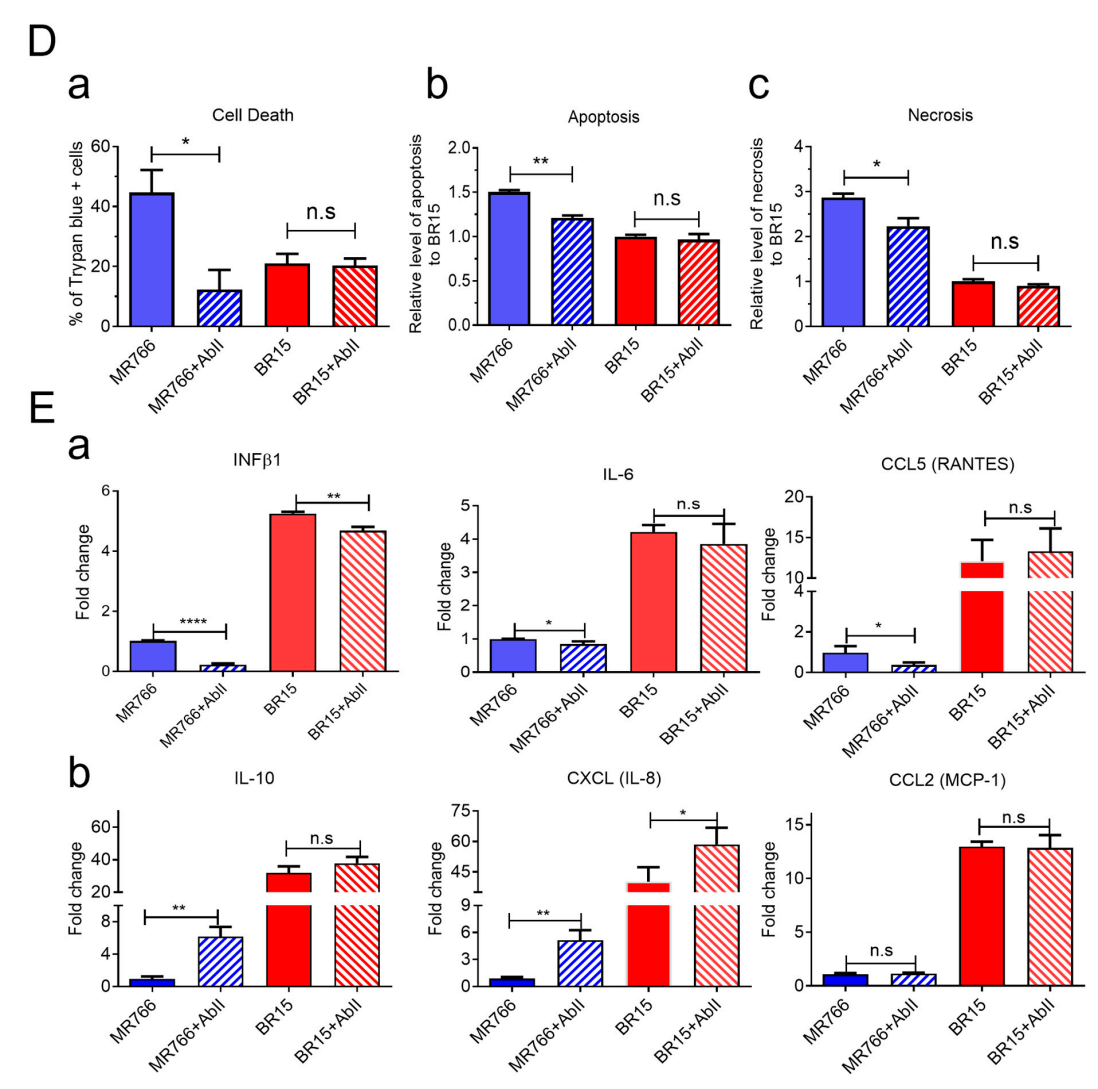
AbII preferentially suppresses MR766-mediated effects but not BR15 in SH-SY5Y cells. (**A**) Images of neurospheres in AbII-treated and untreated groups (**a**) were taken at day 3 *p.i.*, and the numbers (**b**) and sizes of neurospheres (**c**) were quantified in the same way as described in Figure 1. Scale bar in neurosphere images represents 100 µm. AbII preferentially suppresses MR766-mediated effects but not BR15 in neurosphere formation (**A**), viral binding (**B**-**a**), intracellular viral replication (**B**-**b**), viral infection (**C**) as shown by images of immunostaining (**a**) and quantification (**b**), induction of cell death measured by Trypan blue, apoptosis and necrosis (**D**-**a**–**c**), and reduced (**a**) or enhanced (**b**) cellular immune responses of MR766, but no effect on BR15 (**E**). SH-SY5Y cells were infected with BR15 or MR766 ZIKV strains at an MOI of 1.0 in the presence or absence of AbII treatment (2.91 µg/mL). The same methods and timeframe as described in Figure 1 and Figure 3 were used to measure viral binding, intracellular viral replication, cellular immune response and measurement of neurosphere formation. The same method as described in Figure 2 for measuring viral permissiveness was used here to determine the level of viral infection by staining with anti-E mAb 4G2 at 48 h *p.i.* Results of viral replication and apoptotic cell death represent mean ± SD from four independent experiments, each performed in triplicate. Quantitative results are presented as mean ± SD. Statistical significance: * *p* < 0.05; ** *p* < 0.01; **** *p* < 0.0001. n.s., not significant. Scale bar in neurosphere images represents 100 µm.

We next evaluated the efficacy of AbII in preserving neurosphere formation from hNPCs infected with ZIKV. Neurospheres were generated as described earlier, with or without AbII (1:320 dilution, ~2.91 µg/mL), a concentration previously shown to neutralize ZIKV infection in an independent model during mAb screenings. In the presence of AbII, neurosphere integrity was preserved in the MR766 + AbII group, while extensive neurosphere disruption was observed in the untreated MR766-infected control (Figure 4A-a). The quantification revealed that the MR766 + AbII group produced significantly more neurospheres (21.6 ± 1.69) than the untreated MR766 group (14.6 ± 2.86; *p* < 0.05) (Figure 4A-b). Moreover, neurosphere size was significantly increased by AbII treatment, from 140 ± 34.4 µm in the MR766 group to 195.8 ± 44.1 µm in the MR766 + AbII group (Figure 4A-c; *p* < 0.01). In contrast, AbII treatment had no significant impact on BR15-infected neurospheres in terms of morphology, number, or size, compared to untreated BR15 controls (Figure 4A).

To verify this strain-specific discrepancy, we assessed AbII’s effects on viral attachment, replication, cell viability, and apoptotic cell death. Under identical infection conditions, AbII significantly reduced MR766 viral attachment and replication (Figure 4B), infection levels (Figure 4C), and apoptotic cell death (Figure 4D). However, no measurable effects were observed in BR15-infected cells across these assays (Figure 4B–D).

We also examined the influence of AbII on host cellular immune responses. Levels of the same cytokine and chemokine as described in Figure 3 were assessed under the same conditions. Consistent with the lack of effect on BR15 infection, AbII treatment did not significantly alter the levels of IL-1β, IL-6, IL-10, CCL2 (MCP-1), CCL5 (RANTES), or CXCL8 (IL-8) in BR15-infected cells (Figure 4E). Other cytokines or chemokines that did not respond to AbII are listed in Figure S4B.

Together, these findings demonstrate that AbII selectively neutralizes MR766 activity by preserving neurosphere integrity through the reduction in viral attachment, replication, cytotoxicity, and pro-inflammatory responses. However, AbII does not confer comparable protective effects against the contemporary BR15 strain, underscoring the antigenic divergence in E protein epitopes between ancestral and epidemic ZIKV strains.

### 3.5. Molecular Dynamics Simulation of AbII with E Proteins from MR766 and BR15

**Figure 5:**
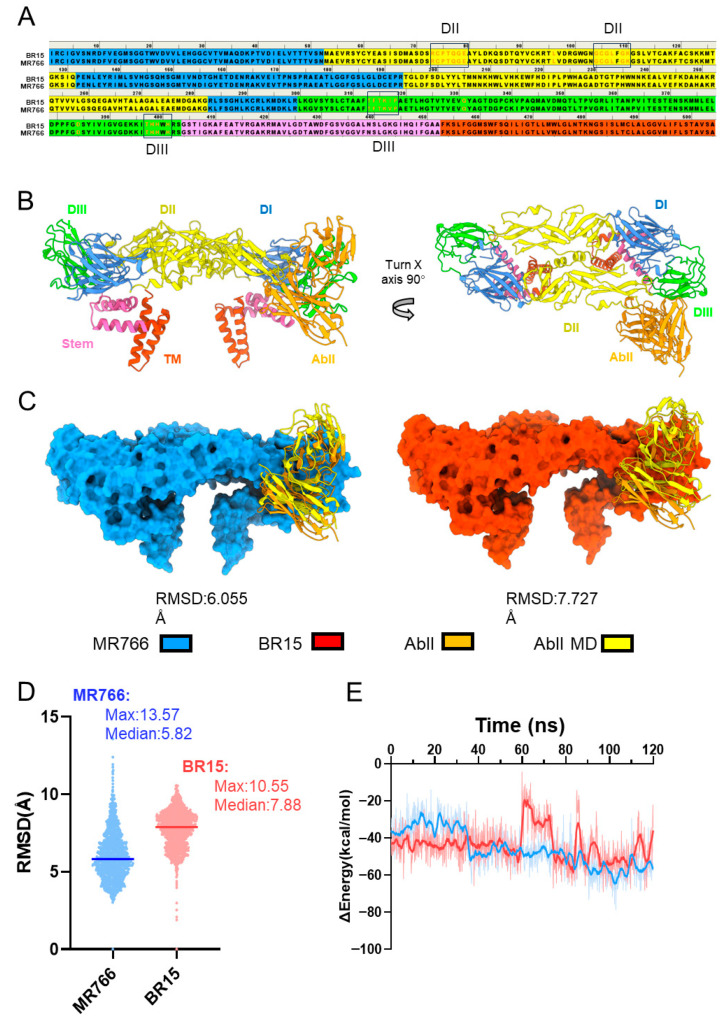
Molecular dynamic simulation of AbII with E protein of BR15 and MR766. (**A**) Sequence alignment of the E protein from two ZIKV strains, MR766 and BR15. Domain I is highlighted in blue, Domain II in yellow, Domain III in green, the stem region in pink, and the transmembrane domain (TM) in red. The epitope recognized by the AbII antibody is indicated in orange text. (**B**) Structure of the E protein dimer in complex with the AbII antibody predicted by AlphaFold3 (AF3). The E protein domains are colored as in (**A**), and the AbII antibody is shown in orange. The antibody primarily binds at the interface between domain II and domain III. An additional structural alignment between our AlphaFold model (MR766 with AbII) and the PDB structures (PDB IDs: 6CO8) is included in Figure S5A. (**C**) Representative average structure of the AbII-bound E protein dimer during the 120 ns molecular dynamics simulation. The average conformation of the AbII is shown in yellow. Structural alignment between the AlphaFold model (MR766 with AbII) and the PDB structures (PDB IDs: 6CO8) is included in Figure S5A. (**D**) This violin plot compares the root mean square deviation (RMSD) distributions of the AbII antibody backbone during molecular dynamics simulations when bound to the E protein of two ZIKV strains, MR766 (blue) and BR15 (red). Each point represents a snapshot from the simulation, and the horizontal lines indicate the median values. (**E**) Binding free energy between the AbII and the E protein from two ZIKV strains (MR766 in blue, BR15 in red) calculated using the Molecular Mechanics Poisson–Boltzmann Surface Area (MMPBSA) method during the 120 ns molecular dynamics simulation. An additional RMSD diagram with ns on the *x*-axis and the integral of the RMSD values calculated is included in Figure S5B.

To understand the structural basis for AbII’s preferential neutralization of MR766 over BR15, we first compared the protein sequences of the two ZIKV strains (Figure 5A). AbII binding sites are highlighted in boxes, showing their localization primarily within domains II (DII, yellow) and III (DIII, green) of the E protein. Sequence comparison revealed no major differences in these binding regions, except for a single amino acid substitution: valine (V) at residue 318 in MR766, which is replaced by isoleucine (I) in BR15. Although both residues are hydrophobic, the subtle difference in side-chain structure may influence local protein dynamics.

Next, we modeled the E protein dimer in a complex with AbII using AlphaFold3 (AF3) [45]. The structural models indicated that AbII interacts primarily with the DII and DIII domains of the E protein, showing strong complementarity at the dimer interface (Figure 5B). These interactions appeared conserved across both MR766 and BR15, suggesting that AbII targets shared conformational epitopes. Despite the conserved binding interface, MD simulations revealed differential complex stability. Over a 120 ns simulation, after aligning the E protein to the initial structure, the AbII antibody in the MR766-AbII complex exhibited a lower average backbone RMSD (6.055 Å) compared to that in the BR15-AbII complex (7.727 Å) (Figure 5C).

This suggests that AbII adopts a more stable binding conformation with MR766. RMSD distribution analysis further showed that the BR15 complex not only had a higher mean value but also a broader spread, reflecting greater structural fluctuations and potentially increased conformational flexibility during AbII binding. In contrast, the MR766 complex exhibited lower, more tightly clustered RMSD values, indicating more stable AbII interactions with this ancestral strain (Figure 5D). To further assess binding strength, we performed Molecular Mechanics Poisson–Boltzmann Surface Area (MM-PBSA) analysis to calculate the binding free energy (ΔG_bind) over the simulation period. More negative ΔG_bind values indicate stronger binding affinity. The MR766-AbII complex consistently showed more favorable (i.e., lower) ΔG_bind values during the first 60 ns, suggesting stronger and more stable interactions than with BR15. Interestingly, BR15 exhibited a biphasic energy profile, with transient increases in ΔG_bind, particularly between 60 and 80 ns, indicating fluctuating and possibly weaker binding interactions during that time (Figure 5E).

Together, these findings demonstrate that while AbII targets conserved E protein epitopes in both MR766 and BR15, its interaction with MR766 is more stable and energetically favorable. This greater stability likely underlies AbII’s preferential neutralization of the MR766 strain.

## 4. Discussion

To investigate virological determinants of increased ZIKV pathogenicity in humans, we compared the ancestral African strain MR766 with two Brazilian epidemic strains, BeH819015 (BR15) and Paraiba01 (ICD). Beyond characterizing viral attachment, infection, and replication, we further examined their effects on hNPC-derived neurosphere formation, a relevant model for studying ZIKV-induced neurogenesis defects and microcephaly [45]. We also assessed the cellular immune responses elicited by these strains and their consequences on neurosphere integrity and cell viability.

Our results reveal striking differences between MR766 and BR15 in their effects on neurosphere formation, viral attachment to neural SH-SY5Y cells, and replication kinetics (Figure 1A–C). These findings align with previous studies reporting strain-specific variations in viral entry and replication in human epithelial and neural cells [38,41]. In agreement, proteomic analyses of ZIKV-infected neurospheres have also shown divergent alterations in pathways related to cell cycle control, apoptosis, and neurogenesis between MR766 and Brazilian strains [45,63].

Critically, we demonstrate that these differences in infectivity and replication correlate directly with neurosphere integrity. MR766 showed greater disruption of neurospheres and higher replication, while BR15 maintained neurosphere integrity to a greater extent. Interestingly, BR15 triggered a stronger host immune response, which may contribute to neuroprotection. Specifically, BR15 induced significantly higher expression of pro-inflammatory cytokines and chemokines, including IL-1β, IL-6, IFNβ1, CCL2 (MCP-1), and CCL5 (RANTES), compared to MR766 (Figure 1D). Despite neither strain significantly altering NF-κB or TNFα expression, both are major inflammatory mediators [54,64], BR15 infection resulted in elevated levels of both pro- and anti-inflammatory cytokines. Notably, BR15 upregulated IL-10 and CXCL8 (IL-8), which, despite their known pro-inflammatory roles, can also exert anti-inflammatory effects depending on cellular context and receptor engagement [65,66,67,68]. These findings suggest that a balanced immune response may mitigate ZIKV-induced cytotoxicity and preserve neurogenesis. The timing and cell-type specificity of cytokine induction may explain discrepancies with earlier reports. For example, one study found no differences in IL-6 and IL-1β between MR766 and BR15 in A549 cells at 24 h *p.i.* [41], whereas we observed distinct profiles at 48 h in neural cells.

Our findings showing that MR766 exhibits stronger neurotropism to human cells than BR15 appear to contradict the common belief that viruses of the Asian lineage are generally more pathogenic to humans than those of the African lineage. To address this apparent paradox, earlier research has indicated that although MR766 can display a strong cytopathic effect in human cells in vitro, its ability to cause severe human diseases is generally considered less potent compared to some of the Asian lineage strains responsible for recent epidemics and congenital Zika syndrome [15]. Therefore, the differences observed in the in vitro and in vivo effects are likely attributed to genetic and functional variations that impact the virus’s ability to infect and cause disease. Moreover, differences in how specific ZIKV strains interact with and evade the immune system may also contribute to variations in disease severity [69]. Another possibility is that MR766 and BR15 may not be fully representative of the African and Asian lineages, respectively. The BR15 strain (BeH819015) was one of the earliest Brazilian ZIKV isolates during the epidemic, and sequence analysis suggests it represents many of the prominent South American ZIKV strains [70]. In contrast, MR766 was the first virus isolated from Africa in 1947, and it causes relatively mild symptoms in humans [4]. While there is a high likelihood that the described differences between these two viral strains are accurate, we cannot make a definitive conclusion about whether such differences exist between the two viral lineages. Testing additional viral strains from both African and Asian lineages is warranted.

Our chimeric virus analyses identified the E protein as the key determinant of differences in viral attachment, replication, neurosphere disruption, and apoptosis between MR766 and BR15. These findings are consistent with our earlier results in epithelial and glioblastoma cells [38], further reinforcing the central role of the E protein in ZIKV neuropathogenesis (Figure 2). Note that, ideally, we would have tested the specific effect of the E protein by swapping only the E protein between MR766 and BR15. However, generating such viral constructs is complex and time-consuming. Instead, we utilized previously generated hybrid viruses from a prior study [38]. Our previous work with these viruses suggested a correlation between the ZIKV E protein and viral attachment. Building upon that foundation, this study further underscores the role of the E protein in modulating immune responses and neuropathogenesis. While this study centers on the E protein, it is important to note that other viral proteins, including both structural and non-structural proteins, likely contribute to lineage-specific differences as well [38,70].

The flavonoid isoquercitrin Q3G demonstrated potent antiviral activity against both MR766 and BR15 strains. Q3G inhibited viral entry, replication, and cytopathic effects and preserved neurosphere integrity, with a moderate therapeutic index of 21.71 (Figure 3). Mechanistically, Q3G suppressed E protein-mediated functions and modulated host immune responses. It reduced levels of pro-inflammatory cytokines (IFNβ1, IL-6, CCL5), while enhancing the expression of anti-inflammatory IL-10 and CXCL8 (IL-8), suggesting a potential dual role in antiviral and neuroprotective responses. Although further studies are required to validate the anti-inflammatory functions of IL-10 and CXCL8 in this context, our data support the hypothesis that Q3G not only limits viral replication but also modulates the immune environment to protect neurogenesis.

In contrast, the monoclonal antibody AbII, developed against the PRVABC59 strain E protein, showed preferential inhibition of MR766 over BR15 across all tested parameters, including neurosphere formation, viral attachment, infection, replication, apoptosis, and immune responses (Figure 4). This result was unexpected, as PRVABC59 belongs to the Asian lineage and is genetically closer to BR15 than MR766. However, the discrepancy may be due to functional differences among Asian lineage strains or the fact that PRVABC59 was isolated from a likely asymptomatic individual, possibly underrepresenting virulent epidemic strains.

Molecular simulations revealed that AbII interacts with conserved epitopes in domains II and III of the E protein in both MR766 and BR15 (Figure 5A,B). However, the BR15-AbII complex exhibited greater structural fluctuations and weaker binding affinity, as indicated by higher RMSD values and less favorable ΔG_bind scores compared to MR766 (Figure 5C–E). These findings suggest that subtle differences in E protein conformation or dynamics may underlie the differential neutralization. Additionally, the concentration of AbII used in our ZIKV infection assays (2.91 µg/mL) was below its in vitro IC_50_ for inhibiting the MR766 E protein (2.95 ± 0.25 µg/mL). While this IC_50_ value against E protein alone may not directly translate to effective antiviral concentrations, the suboptimal antibody level used in our assays likely contributed to the limited neutralization observed. Therefore, it is plausible that AbII could also neutralize BR15 at higher concentrations. To address this, a dose–response curve of AbII against BR15 infection could be generated, which is a potential experiment we plan to explore in future studies.

## 5. Conclusions

This study highlights fundamental differences in the virological, neuropathological, and immunological properties of African and epidemic ZIKV strains. We show that the E protein is a key driver of strain-specific pathogenicity, and that its targeting by therapeutic agents can yield distinct outcomes. While Q3G is effective against both strains through inhibition of viral entry and immune modulation, AbII exhibits strain-selective neutralization against MR766. Together, our findings provide mechanistic insights into ZIKV neuropathogenesis and support the development of targeted therapeutics to combat diverse viral strains.

## Data Availability

The original contributions presented in this study are included in the article. Further inquiries can be directed to the corresponding author.

## Supplementary Materials

The following supporting information can be downloaded at https://www.mdpi.com/article/10.3390/pathogens14070716/s1, Figure S1: Contrasting effect of MR766 and BR15; Figure S2: Effect of chimeric ZIKV infection; Figure S3: Cytokines and chemokines that did not respond to Q3G treatment; Figure S4: IC50 of anti-E protein and cytokines and chemokines that did not respond to AbII treatment; Figure S5: Additional alignment of AlphaFold model of MR766 with AbII and RMSD diagram; Table S1: PCR primers used in this study.

## Abbreviations

The following abbreviations are used in this manuscript:

Adv	Adenovirus
CC_50_	50% Cytotoxic Concentration
cDNA	Complementary DNA
CHIKV	Chikungunya Virus
CMC	Carboxymethylcellulose
CNS	Central Nervous System
DENV	Dengue Virus
DMEM	Dulbecco’s Modified Eagle’s Medium
DMSO	Dimethyl Sulfoxide
E	Envelope protein
EC_50_	Half-maximal Effective Concentration
FBS	Fetal Bovine Serum
GAPDH	Glyceraldehyde 3-phosphate dehydrogenase
GBS	Guillain–Barré Syndrome
hNPC	Human Neural Progenitor Cell
IC_50_	Half-maximal Inhibitory Concentration
JEV	Japanese Encephalitis Virus
mL	Milliliter
MOI	Multiplicity of Infection
nAbs	Neutralizing Antibodies
*p.i.*	Post Infection
PBS	Phosphate-buffered saline
PFU	Plaque-forming unit
Q3G	quercetin-3-β-O-D-glucoside
ssRNA	Single-Stranded RNA
WNV	West Nile Virus
ZIKV	Zika Virus
