# Supplementary material for: Distinct Virologic Properties of African and Epidemic Zika Virus Strains: The Role of the Envelope Protein in Viral Entry, Immune Activation, and Neuropathogenesis
Source: Pathogens. 2025 Jul 19;14(7):716. doi: 10.3390/pathogens14070716 (PMC12298065; doi:10.3390/pathogens14070716)
Supplement: Supplementary file 1 [file pathogens-14-00716-s001.zip › pathogens-3722830-supplementary/Legend of Supplemental Figures.docx]

**Legend of Supplemental Figures**

**Figure S1.** Contrasting effect of MR766 and BR15. Supplemental data to **Figure 1**: (**A**) Contrasting effect of MR766 and BR15 on the formation of neurosphere with increased MOI at day 4 *p.i*. Representative images of neurosphere formation are shown in **Figure 1C**. Additional numbers of neurospheres are shown here by MOI 5 and 10 of MR766 and BR15 ZIKV strains. Note that infection with MR766 at MOI 10 results in complete destruction of neurosphere formation. (**B**) Cytokines and Chemokines did not respond to ZIKV infection. Neuroblastoma SH-SY5Y cells were infected with MR766 or BR15 with MOI of 1.0. Infected cells were collected at 48 hours p.i. The levels of these markers were measured by qRT-PCR.

**Figure S2.** Effect of chimeric ZIKV infection. Supplemental data to **Figure 2**: Effect of chimeric ZIKV infection on neurospheres formation at three days *p.i*. with ZIKV strains MR766, BR15, and chimeric strains (M/B and B/M), or uninfected controls (Mock). Representative images of neurosphere development are shown in **Figure 2B**. Additional numbers of neurospheres are shown here.

**Figure S3.** Supplemental data to **Figure 3E**: Cytokines and chemokines did not respond to Q3G treatment. Neuroblastoma SH-SY5Y cells were infected with MR766 or BR15 with MOI of 1.0 in the presence or absence of Q3G (12.5 μM). Infected cells were collected at 48 hours p.i. The levels of these markers were measured by qRT-PCR.

**Figure S4.** Supplemental data to **Figure 4**: **(A)** Determination of IC50 value of AbII against Adv-E-induced cell death at MOI of 1,000. **(B)** Cytokines and chemokines did not respond to AbII treatment. Neuroblastoma SH-SY5Y cells were infected with MR766 or BR15 with MOI of 1.0 in the presence or absence of AbII treatment (2.91 µg/mL). Infected cells were collected at 48 hours p.i. The levels of these markers were measured by qRT-PCR.

**Figure S5.** Supplemental data to **Figure 5**: **(A)** Structural alignment between our AlphaFold model (MR766 with AbII) and the PDB structures (PDB IDs: 6CO8). The structural alignment analysis revealed an RMSD of 2.622 Å between the AlphaFold-predicted structure and the experimentally resolved Zika protein E (PDB), suggesting substantial conformational agreement and reflecting strong overall topological conservation between the computational and experimental models. (**B**) Time-based RMSD plots of backbone fluctuations of the AbII antibody during molecular dynamics simulations. The time-based RMSD plots of backbone fluctuations of the AbII antibody during molecular dynamics were simulated by aligning the E protein of two Zika virus strains, MR766 (blue) and BR15 (red).
