# Supplementary material for: Distinct Virologic Properties of African and Epidemic Zika Virus Strains: The Role of the Envelope Protein in Viral Entry, Immune Activation, and Neuropathogenesis
Source: Pathogens. 2025 Jul 19;14(7):716. doi: 10.3390/pathogens14070716 (PMC12298065; doi:10.3390/pathogens14070716)
Supplement: Supplementary file 1 [file pathogens-14-00716-s001.zip › pathogens-3722830-supplementary/Table S1.docx]

| **Gene** | **Primer (5’ to 3’)** |
| --- | --- |
| NF-κB | Forward: ACGAATGACAGAGGCGTGTA |
|  | Reverse: TAGCTCTTTTTCCCGATCTCCC |
| TNFα | Forward: CGAGTGACAAGCCTGTAGC |
|  | Reverse: GGTGTGGGTGAGGAGCACAT |
| IL-6 | Forward: AATAACCACCCCTGACCCAAC |
|  | Reverse: AATCTGAGGTGCCCATGCTAC |
| IL-1β | Forward: AGCTGATGGCCCTAAACAGATGAA |
|  | Reverse: AAGCCCTTGCTGTAGTGGTGGT |
| INFβ1 | Forward: GGCAGTATTCAAGCCTCCCAT |
|  | Reverse: TCTCCTGTTGTGCTTCTCCAC |
| CCL2 | Forward: CCCTTCTGTGCCTGCTGCTC |
|  | Reverse: CACTTGCTGCTGGTGATTCTTCTAT |
| CCL5 | Forward: CCAGCAGTCGTCTTTGTCAC |
|  | Reverse: CTCTGGGTTGGCACACACTT |
| CXCL8 | Forward: TCTTGGCAGCCTTCCTGATTTC |
|  | Reverse: AGGTTTGGAGTATGTCTTTATGCAC |
| IL-10 | Forward: GACTTTAAGGGTTACCTGGGTTG |
|  | Reverse: TCACATGCGCCTTGATGTCTG |
| IL-12 | Forward: TAGGGACCTGAGATGCTATCG |
|  | Reverse: CCCGGAGCTAAGGCAACAC |
| IL-17 | Forward: GCTGTCGATATTGGGGCTTG |
|  | Reverse: GGAAACGCGCTGGTTTTCA |
| IL-18 | Forward: TCTTCATTGACCAAGGAAATCGG |
|  | Reverse: TCCGGGGTGCATTATCTCTAC |
| IFNγ | Forward: AGCAGGAAGTCGATTATGATCCC |
|  | Reverse: CTGGCACTGAATCTCGTCACA |
| IL-4 | Forward: CCAACTGCTTCCCCCTCTG |
|  | Reverse: TCTGTTACGGTCAACTCGGTG |
| CCL3 | Forward: CTCCTCTGCACCATGGCTCTC |
|  | Reverse: GCTCGTCTCAAAGTAGTCAGCTATG |
| CXCL12 | Forward: ATTCTCAACACTCCAAACTGTGC |
|  | Reverse: ACTTTAGCTTCGGGTCAATGC |
| TGFβ | Forward: GGCCAGATCCTGTCCAAGC |
|  | Reverse: GTGGGTTTCCACCATTAGCAC |

**Table S1.** Primers used in this study.
