# Supplementary figures and images for: Distinct Virologic Properties of African and Epidemic Zika Virus Strains: The Role of the Envelope Protein in Viral Entry, Immune Activation, and Neuropathogenesis
Source: Pathogens. 2025 Jul 19;14(7):716. doi: 10.3390/pathogens14070716 (PMC12298065; doi:10.3390/pathogens14070716)

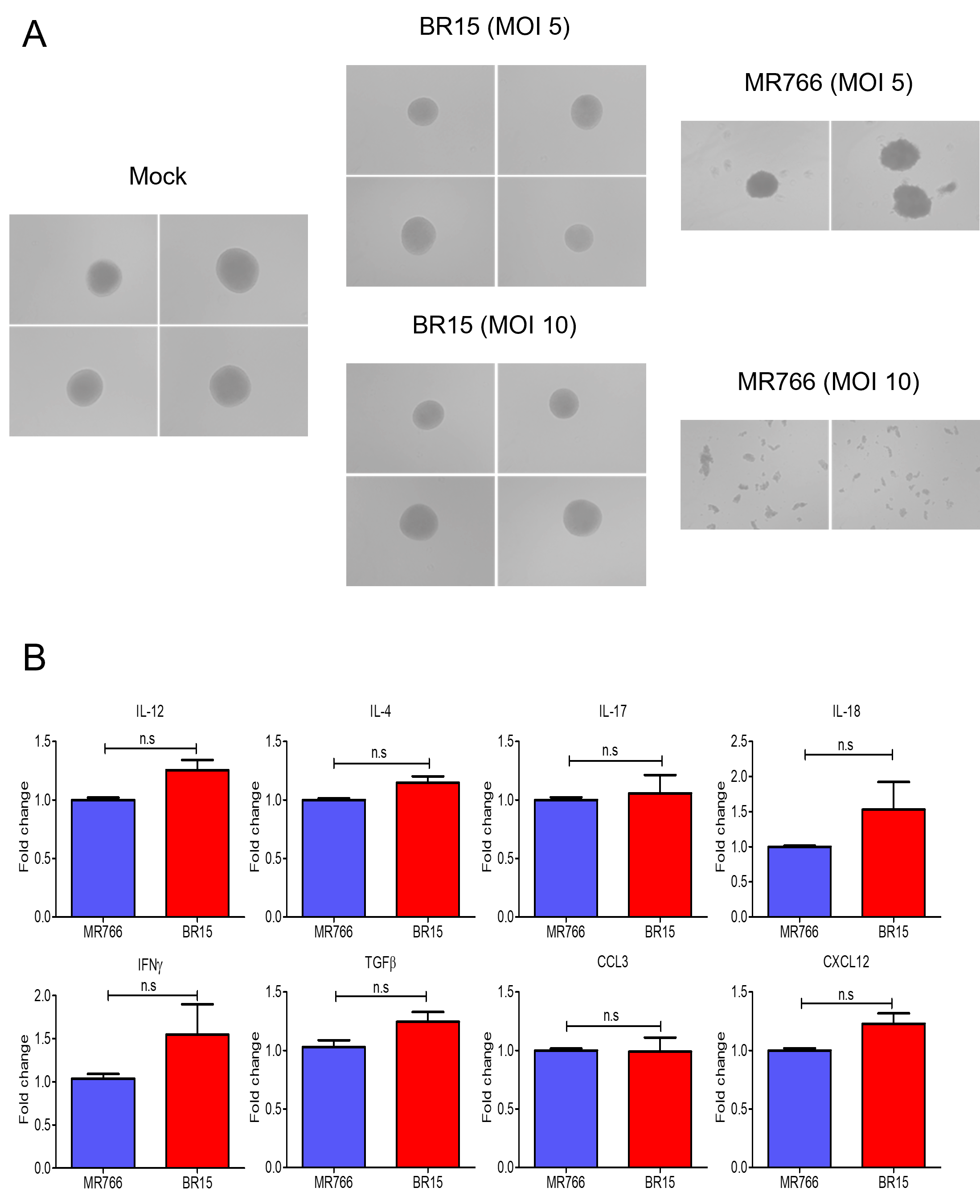

Supplement: Supplementary file 1 [file pathogens-14-00716-s001.zip › pathogens-3722830-supplementary/Figure S1.tif]

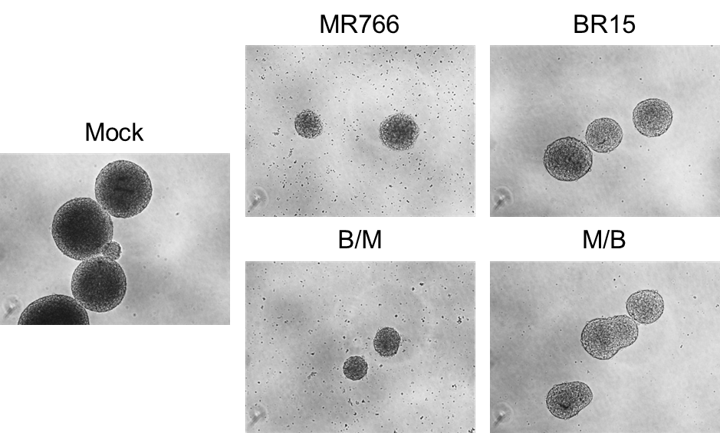

Supplement: Supplementary file 1 [file pathogens-14-00716-s001.zip › pathogens-3722830-supplementary/Figure S2.tif]

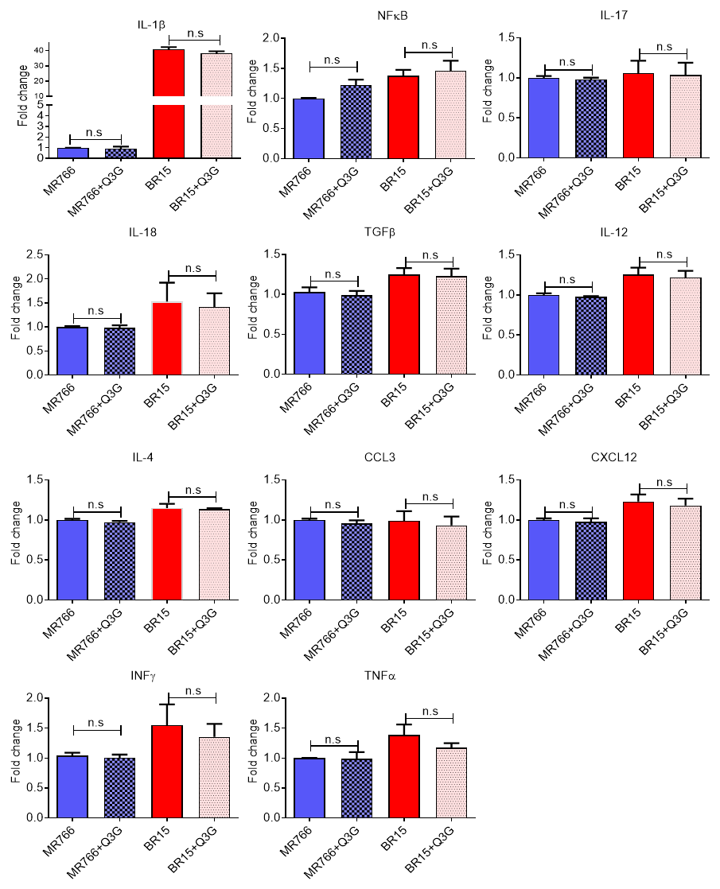

Supplement: Supplementary file 1 [file pathogens-14-00716-s001.zip › pathogens-3722830-supplementary/Figure S3.tif]

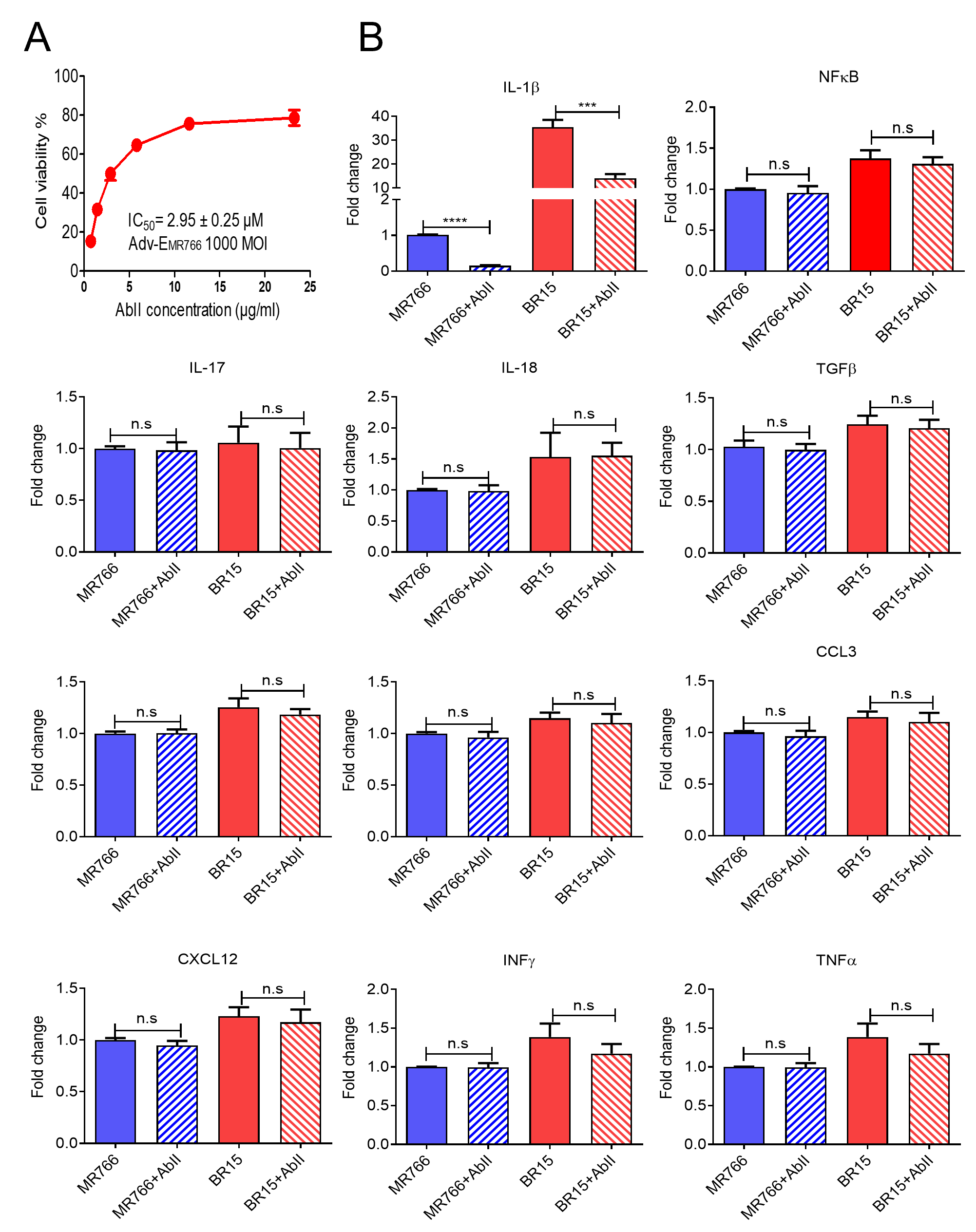

Supplement: Supplementary file 1 [file pathogens-14-00716-s001.zip › pathogens-3722830-supplementary/Figure S4.tif]

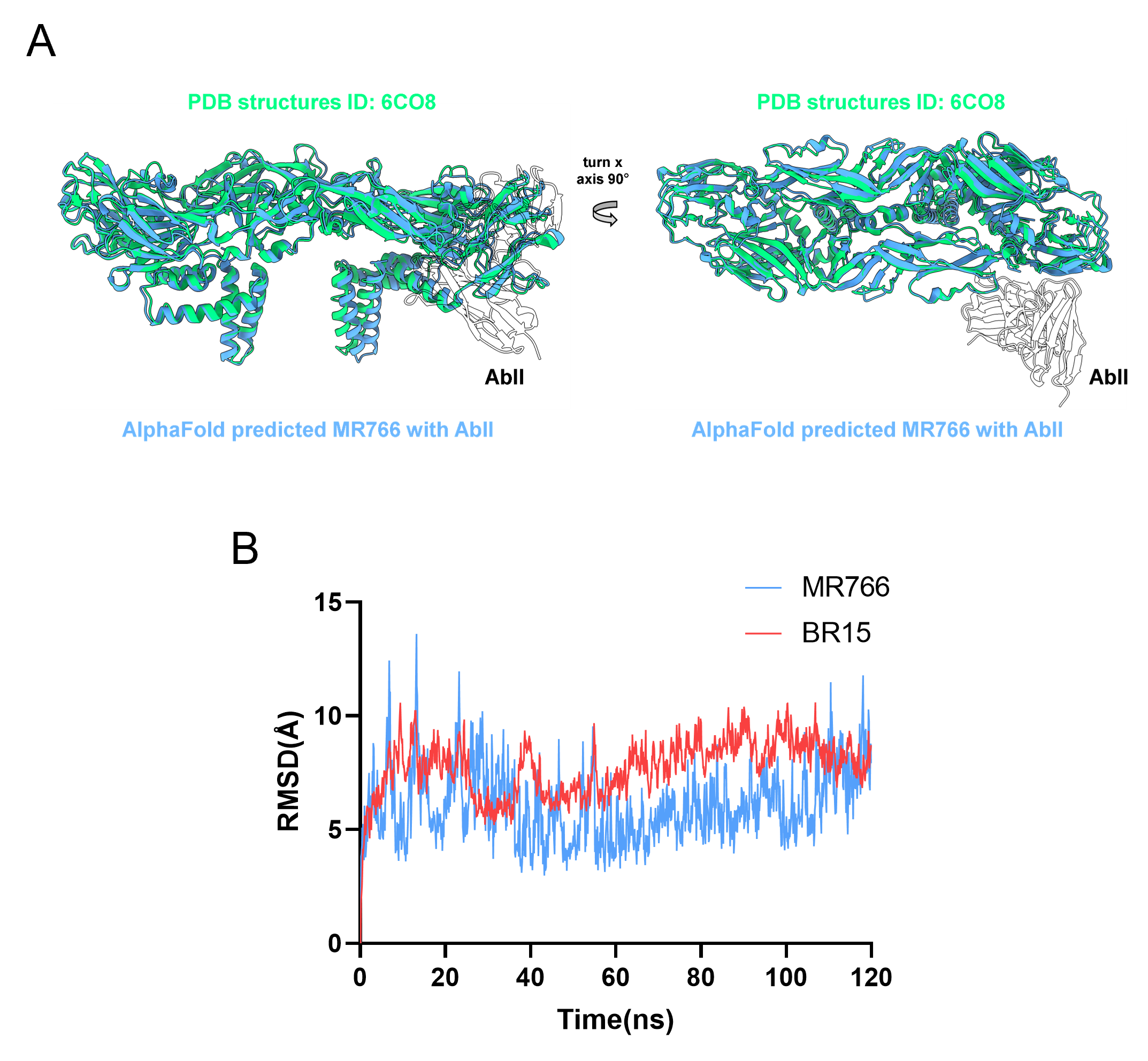

Supplement: Supplementary file 1 [file pathogens-14-00716-s001.zip › pathogens-3722830-supplementary/Figure S5.tif]
